# Arthritis has an impact on the daily lives of Canadians young and old: results from a population-based survey

**DOI:** 10.1186/s12891-015-0691-2

**Published:** 2015-08-30

**Authors:** Siobhan O’Donnell, Corneliu Rusu, Gillian A. Hawker, Sasha Bernatsky, Louise McRae, Mayilee Canizares, Crystal MacKay, Elizabeth M. Badley

**Affiliations:** Centre for Chronic Disease Prevention, Public Health Agency of Canada, Ottawa, ON Canada; Department of Medicine, University of Toronto School of Medicine, Toronto, ON Canada; Department of Medicine, Women’s College Hospital, Toronto, ON Canada; Division of Rheumatology and Clinical Epidemiology, McGill University Health Centre, Montreal, QC Canada; Division of Clinical Epidemiology, Research Institute of the McGill University Health Centre, Montreal, QC Canada; Arthritis Community Research and Evaluation Unit, Division of Health Care and Outcomes Research, Toronto Western Research Institute, Toronto, ON Canada; Dalla Lana School of Public Health, University of Toronto, Toronto, ON Canada

## Abstract

**Background:**

There is a perception that the impacts of arthritis are greatest among older adults. However, the effect of age on health-related outcomes in individuals with arthritis has not been explicitly studied. This study examined whether the physical and mental health impacts of arthritis are greater in older (75+ years) versus younger (20–44, 45–64 and 65–74 years) Canadian adults.

**Methods:**

Data were from the arthritis component of the 2009 Survey on Living with Chronic Diseases in Canada. The responses were weighted to be representative of Canadians (≥20 years) with arthritis. Associations between age and the prevalence of severe/frequent joint pain, severe/frequent fatigue, sleep limitations, instrumental activities of daily living (IADLs) limitations, high levels of stress, suboptimal general and suboptimal mental health, were examined descriptively prior to conducting multivariate log-binomial regression analyses.

**Results:**

A total of 4565 respondents completed the survey (78 % response rate). Individuals with arthritis were mostly female (63 %), of working age (57 %) and overweight or obese (67 %). Upon adjusting for covariates, younger (20–44 years) and/or middle aged (45–64 years) adults were more likely than those older (75+ years) to report severe/frequent joint pain, sleep limitations, high levels of stress and suboptimal mental health. After adjusting for covariates, age was not associated with IADL limitations, severe/frequent fatigue or suboptimal general health.

**Conclusions:**

Contrary to the belief that older adults with arthritis experience more severe physical and mental health outcomes, we found that older adults were less likely to report worse outcomes than younger adults. In light of these findings, public health messaging should stress that arthritis does not just affect the elderly and emphasize the importance of timely diagnosis and management at all ages in order to prevent or, minimize arthritis-related impairment.

## Background

Arthritis encompasses more than 100 rheumatic diseases and conditions that affect the joints, the tissues that surround the joint and other connective tissue [1]. It is one of the most common chronic conditions and a leading cause of disability in North America [1–3]*.* The effects of arthritis include joint pain, fatigue, reduced mobility, limitations in self-care, domestic life and restriction in various life situations such as leisure activities and the labour force. Due in large part to these effects, arthritis is also associated with impaired mental health. All of these problems not only affect the individual living with arthritis, but also their families and informal carers [4–8].

The prevalence of arthritis increases with age as do the ensuing impacts including pain, limitations in activities of daily living, and other aspects of health [9–12]. The age-related increase in prevalence, coupled with the fact that joint damage accumulates with disease duration [11], has led to a wide-spread belief that the impact of arthritis is greatest in older individuals. However, the effect of age on health-related outcomes in individuals with arthritis has not been explicitly studied. Gaining a better understanding of the effects of age on health related outcomes among those living with arthritis may help to develop more targeted public health messaging as well, may facilitate more timely diagnosis and treatment among those living with the condition.

We used data from a representative national sample of Canadian adults aged 20 years and older with arthritis [13, 14], to investigate whether the physical and mental health impacts of arthritis are greater in older (75+ years) versus younger Canadian adults (20–44, 45–64 and 65–74 years).

## Methods

### Data source

The arthritis component of the 2009 Survey on Living with Chronic Disease in Canada (SLCDC), a cross-sectional follow-up survey to the 2008 Canadian Community Health Survey (CCHS), was developed by the Public Health Agency of Canada and Statistics Canada in collaboration with external arthritis experts [13, 14]. The survey was designed to provide new information related to the experiences of Canadians with arthritis (all types) and the approaches that they and their health professionals use to manage their condition. The SLCDC was administered by trained personnel via a structured telephone interview (English or French) in February and March 2009. To confirm respondents had diagnosed arthritis, it includes the questions ‘To begin, do you have arthritis, excluding fibromyalgia, that has been diagnosed by a health professional?’ (Response options: "yes", "no"). This is followed by ‘Do you know the kind of arthritis you have?’ (Response options: "yes", "no"); and, “What kind of arthritis do you have?” (with a list of 14 possible responses and instructions to mark all that apply).

### Target population and sample selection

Respondents aged 20 years or older who reported having been diagnosed with arthritis by a health professional as part of the 2008 CCHS^1^ were eligible for participation in the 2009 SLCDC [14]. Residents of the three territories, persons living on Indian Reserves or Crown lands, residents of institutions, full-time members of the Canadian Armed Forces and residents of certain regions were excluded from the sampling frame. These exclusions represent two percent of the overall Canadian population.

A total of 7062 respondents ages 20 years who reported having diagnosed arthritis were selected from the source survey. Upon determining the out-of-scope respondents (because of death, moving outside of Canada, not having the chronic condition, etc.), 5820 respondents were deemed eligible and contacted to take part. The SLCDC was designed to produce reliable estimates at the national level by sex and the following targeted age groups: 20 to 44 years, 45 to 64 years, 65 to 74 years and 75 years and older.

### Ethics

Respondent’s participation in the SLCDC was completely voluntary and proxy interviews were not permitted [14]. Statistics Canada is prohibited by law from releasing any information it collects which could identify any person, business, or organization, unless consent has been given by the respondent or as permitted by the *Statistics Act*^2^. Share partners, including the Public Health Agency of Canada, have access to the data under the terms of the data sharing agreements. These data files contain only information on respondents who agreed to link their information from the SLCDC to their responses from the CCHS and share their data with Statistics Canada’s partners. Personal identifiers are removed from the share files to respect respondent confidentiality. Users of these files must first certify that they will not disclose, at any time, any information that might identify a survey respondent.

### Measures

The SLCDC survey content was developed through an ongoing consultative process between the Public Health Agency of Canada, Statistics Canada, and external arthritis experts, both clinicians and researches [14]. The survey questions selected were gleaned from the peer-reviewed literature and/or publically available population-based surveys including the CCHS conducted by Statistics Canada and the Arthritis Conditions Health Effects Survey (ACHES) conducted by the Centers for Disease Control and Prevention in the US.

#### Impact-of-arthritis outcomes

included joint pain, fatigue, sleep limitations, instrumental activities of daily living (IADLs) limitations, self-rated general health, life stress and mental health.

#### Joint pain

The intensity of a respondent’s joint pain (in general) in the past month was assessed by asking on average, how bad their joint pain was out of ten, with one representing “little” and ten representing “as bad as it could be”. The frequency of their joint pain in the past month was classified as “always”, “often”, “sometimes”, “rarely”, or “never”. For analytical purposes, severe joint pain was defined as pain intensity seven or more out of ten and was considered frequent if experienced “always” or “often” [15, 16].

#### Fatigue

The intensity and frequency of respondents’ symptoms of fatigue were assessed in an identical manner to pain with severe fatigue defined as fatigue intensity seven or more out of ten and was considered frequent if experienced “always” or “often” [15, 16].

#### Sleep limitations

Participants reported limitations in getting a good night sleep due to arthritis in the past month as: “a lot”, “a little”, or “not at all”.

#### IADLs limitations

Respondents’ limitations in 1) bathing or dressing; 2) getting around the house; 3) doing household chores; 4) running errands or shopping; and 5) participating in recreation, leisure, hobbies or social activities due to their arthritis in the past month were categorized as: “a lot”, “a little”, or “not at all”. Severe limitations in IADLs were defined as being limited “a lot” in three or more of the five aforementioned IADLs [17].

#### General health

Respondents classified their general health as “excellent”, “very good”, “good”, “fair”, or “poor”. Suboptimal general health was defined as having “fair” or “poor” general health [18].

#### Life stress

The amount of stress a respondent experienced on a daily basis was categorized as “not at all stressful”, “not very stressful”, “a bit stressful”, “quite a bit stressful”, or “extremely stressful”. A high level of stress in life was defined by most days being “quite a bit stressful” or “extremely stressful”.

#### Mental health

was ascertained in an identical manner to general health, with a report of “fair” or “poor” denoting suboptimal mental health [19].

#### Covariates

that have been shown in other studies to affect the severity of arthritis outcomes [20–24] were selected *a priori* and included age, sex, body mass index (BMI), education, type of arthritis, number of painful joints, disease duration and number of other chronic conditions. Age was defined as the age at the time of the interview and categorized into one of the aforementioned age groups: 20 to 44, 45 to 64, 65 to 74 and 75+ years. Respondents were classified as overweight or obese based on the World Health Organization (WHO) standards for BMI calculated from reported weight and height: BMI of 25–29.9 kg/m^2^ or >30 kg/m^2^, respectively [25]. Highest level of education attained was classified into one of two categories: less than post-secondary and post-secondary. Type of arthritis was classified into one of four subgroups: 1. osteoarthritis (OA); 2. inflammatory and other types of arthritis; 3. inflammatory and other types of arthritis as well as, OA; and 4. does not know type of arthritis (where "inflammatory and other types of arthritis" included rheumatoid arthritis, ankylosing spondylitis, gout, lupus, polymyalgia rheumatica, polymyositis, psoriatic arthritis, Reiter’s syndrome, scleroderma/systemic sclerosis, Sjogren’s syndrome, vasculitis, fibromyalgia, and bursitis/carpal tunnel/tendonitis). Number of painful joint sites (with 19 response options including neck, back, right or left shoulder, elbow, wrist, hand/fingers/thumb, hip, knee, ankle, foot/toes or other) in the past month was classified as polyarticular joint pain when respondents reported four or more painful joints in the past month [26]. Disease duration (i.e., the number of years living with an arthritis diagnosis) was classified into one of two categories: <10 years or ≥10 years [9]. Lastly, the number of other chronic conditions (0, 1, 2, 3+) was determined based on self-reported diagnosis of the following chronic conditions: asthma, diabetes, heart disease, stroke, cancer, chronic obstructive pulmonary disease, back problems (excluding arthritis), bowel disorder/Crohn’s disease/colitis, Alzheimer’s disease/other dementia and mood/anxiety disorders.

### Statistical analysis

Estimates were weighted using survey weights^3^ generated by Statistics Canada in order to reflect the number of individuals in the Canadian population (aged ≥20 years) with arthritis [14]. The 95 % confidence intervals (CIs) were derived using exact standard errors generated through the bootstrap re-sampling method to account for the complex sampling design [27]. Bootstrap techniques are commonly used to obtain appropriate variance estimates from studies using complex designs, since traditional estimation methods are inappropriate in the presence of clustering, stratification, and unequal selection probabilities [28].

Descriptive analyses were performed to describe the proportion of individuals with arthritis reporting the specified impact-of-arthritis outcomes by age group. Multivariate log-binomial regression models were used to estimate the adjusted prevalence ratios for the association between age and the prevalence of severe/most limited/poor/most limited/poor impact-of-arthritis outcomes controlling for sex, BMI, education, type of arthritis, number of painful joints, disease duration and number of other chronic conditions [29, 30]. Tests for collinearity were negative and missing data counted for less than 10 % of the original data in all models. All analyses were performed with SAS Enterprise Guide version 5.1 (SAS Institute, Cary, NC).

## Results

Of the 5820 eligible participants, 4565 (78.4 %) responded to the survey and consented to share their data with the Public Health Agency of Canada, Health Canada and provincial governments and allowed linkage of their 2009 SLCDC information to their 2008 CCHS responses [14]. The majority of individuals with arthritis were female (63.1 %), of working age (12.5 % were 20–44 years of age, and 47.0 % were 45–64), overweight or obese (39.9 % were overweight, and 27.4 % were obese), post-secondary educated (68.7 %), had four or more painful joints (55.2 %), were diagnosed with arthritis over 10 years ago (50.3 %) and had at least one other chronic condition (36.3 %). A large proportion did not know the type of arthritis that they have (43.2 %) (Table 1).

**Table 1:** Socio-demographic and clinical characteristics in individuals with arthritis (*n* = 4565)

Characteristics	Number	% (95 % CI)^a^
Sex (female)	2886	63.1 (61.4–65.0)
Age group (years)		
20–44	488	12.5 (11.2–13.8)
45–64	1748	47.0 (45.1–48.9)
65–74	1227	22.0 (20.5–23.6)
75+	1102	18.5 (17.3–19.7)
BMI (kg/m^2^)		
Underweight or normal (<25.0)	1597	32.7 (30.4–35.1)
Overweight (25.0–29.9)	1674	39.9 (37.1–42.7)
Obese (>30.0)	1195	27.4 (25.0–29.8)
Post-secondary education	2676	68.7 (66.4–71.1)
Type of arthritis		
OA	1755	38.0 (35.3–40.7)
Inflammatory and other types of arthritis	646	16.3 (14.3–18.2)
Inflammatory and other types of arthritis plus OA	142	2.5 (1.8–3.3)
Does not know type of arthritis	1891	43.2 (40.2–46.2)
Multiple (≥4) painful joints	2581	55.2 (52.6–57.9)
Disease duration (≥10 years)	2316	50.3 (47.6–53.1)
Number of other chronic conditions		
0	1282	28.8 (26.3–31.2)
1	1485	36.3 (33.0–39.7)
2	916	19.0 (17.1–20.9)
3+	634	15.9 (13.7–18.1)

2009 Survey on Living with Chronic Diseases, Arthritis Component

^**a**^Prevalence estimates (%) and 95 % confidence intervals (95 % CI) are based on weighted data; *BMI* body mass index; *OA osteoarthritis*

Severe and frequent fatigue was reported more frequently by those within the youngest age group (20–44 years) compared to those aged 75 years or older. Severe and frequent joint pain and being limited a lot in getting a good night sleep due to arthritis was reported more frequently by middle aged (45–64 years) adults compared to those 75 years and older. Suboptimal general health was reported more frequently by those 75+ years or older compared to younger adults aged 20–44 years of age. High levels of life stress and suboptimal mental health was reported more frequently by younger (20–44 years) and middle aged (45–64 years) adults compared to those 75 years or older. There were no significant age group differences among those being limited a lot in at least three IADLs (Table 2).

**Table 2:** Impact-of-arthritis outcomes in individuals with arthritis by age group (*n* = 4565)

Outcomes	Overall		Age group (years)			
			20–44	45–64	65–74	75+
	Number	% (95 % CI)^a^	% (95 % CI)^a^	% (95 % CI)^a^	% (95 % CI)^a^	% (95 % CI)^a^
Joint pain						
(severe and frequent)	1103	26.0 (23.6–28.4)	23.1 (17.2–28.9)	28.5 (24.1–32.9)**	26.7 (21.8–31.6)	20.8 (16.8–24.9)
Fatigue						
(severe and frequent)	804	19.8 (17.4–22.3)	28.8 (22.3–35.3)***	21.8 (17.3–26.2)	13.6 (10.5–16.7)	16.1 (12.5–19.8)
Sleep						
(limited a lot)	1037	24.4 (21.9–27.0)	24.2 (18.5–30.0)	28.8 (24.0–33.6)***	20.7 (16.5–24.9)	18.0 (14.0–21.9)
IADLs						
(≥3 IADLs limited a lot)	504	11.0 (9.3–12.8)	11.0 (6.6–15.4)^b^	11.0 (8.0–14.0)	10.0 (7.3–12.7)	12.2 (9.1–15.4)
General health						
(fair or poor)	1334	30.4 (27.6–33.2)	22.5 (17.1–27.8)**	30.5 (25.6–35.4)	32.4 (27.9–36.9)	33.0 (27.9–38.0)
Life stress						
(quite a bit or extremely stressful)	862	20.5 (18.5–22.5)	31.6 (25.0–38.1)***	25.4 (21.7–29.0)***	12.1 (9.3–14.9)	10.6 (7.7–13.5)
Mental health						
(fair or poor)	428	11.8 (9.3–14.3)	12.5 (8.3–16.6)^b^***	17.2 (12.3–22.9)***	6.2 (3.9–8.4)^b^	4.4 (2.9–5.9)^b^

2009 Survey on Living with Chronic Diseases, Arthritis Component

^**a**^Prevalence estimates (%) and 95 % confidence intervals (95 % CI) are based on weighted data; *IADLs* Instrumental Activities of Daily Living; ^b^High sampling variability (the coefficient of variation is between 16.6 and 33.3 %); Statistically significantly different from those in the oldest age group (75+ years): *p < 0.05; **p < 0.01; ***p < 0.001

Upon controlling for differences in sex, BMI, education, type of arthritis, number of painful joints, disease duration and number of other chronic conditions, middle age adults (45–64 years) were significantly more likely to report severe and frequent pain compared to those older (75+ years). Furthermore, younger (20–44 years) and middle aged (45–64 years) adults were significantly more likely than those older (75+ years) to being limited a lot in getting a good night sleep, high level of stress, and suboptimal mental health. Age was not associated with severe and frequent fatigue, being limited a lot in three or more IADLs or suboptimal general health (Table 3).

**Table 3:** Association between age and the prevalence of severe/most limited/poor impact-of-arthritis outcomes in individuals with arthritis (*n* = 4565)

	Prevalence Ratio (95 % CI)^a^						
	Joint pain (severe and frequent)	Fatigue (severe and frequent)	Sleep (limited a lot)	IADLs (≥3 ADLs limited a lot)	General health (fair or poor)	Life stress (quite a bit or extremely stressful)	Mental health (fair or poor)
Age group (years)							
20–44	1.3	1.6	1.6	0.7	0.8	3.3	4.1
	(0.9–1.9)	(0.9–2.8)	(1.0–2.6)*	(0.3–1.6)	(0.5–1.2)	(2.0–5.5)***	(1.8–9.4)***
45–64	1.3	1.6	1.5	1.2	0.8	2.5	3.8
	(1.0–1.8)*	(1.0–2.5)	(1.1–2.0)*	(0.7–1.9)	(0.6–1.1)	(1.7–3.6)***	(2.0–7.0)***
65–74	1.2	1.0	1.1	0.8	0.8	1.0	1.5
	(0.9–1.6)	(0.6–1.5)	(0.7–1.5)	(0.5–1.3)	(0.6–1.1)	(0.7–1.5)	(0.7–3.0)
75+	1.0	1.0	1.0	1.0	1.0	1.0	1.0

2009 Survey on Living with Chronic Diseases, Arthritis Component

^**a**^Prevalence ratios and 95 % confidence intervals (95 % CI) are adjusted for sex, body mass index, level of education, type of arthritis, multiple (≥4) painful joints, disease duration, and number of other chronic conditions and are based on weighted data; *IADL* Instrumental Activities of Daily Living; *p < 0.05; **p < 0.01; ***p < 0.001

## Discussion

Contrary to the expectation that older individuals with arthritis experience more severe outcomes, we found (after controlling for sex, BMI, education, type of arthritis, number of painful joints, disease duration and other chronic conditions) that those in the oldest age group (75+ years) were less likely to report worse outcomes, such as joint pain, sleep limitations, stress and mental health than their younger counterparts. These findings are in line with previous national surveys showing that the proportion of individuals with arthritis who report activity limitations and worse self-rated health is significantly greater than individuals with no chronic conditions as well as individuals with other chronic conditions [1, 31].

While there are few studies which explicitly address the effect of age on arthritis outcomes, some indication of the effect can be gleaned from population-based studies which also include age in the tables of results. In our study, older adults (75 years or older) were more likely to report poorer general health than younger adults (20–44 years of age); however, after controlling for various factors, age was not associated with suboptimal general health. Data from some US population health surveys also did not find a clear age gradient in self-reported general health [32, 33]. For instance, data from the US National Health Measurement Study showed no age-gradient in health-related quality of life indices which combine both physical functioning and mental health [33]. In contrast, other studies have found that reporting only fair to poor health increases with age [22, 34]; however, unlike our study, those data were not comprehensively adjusted for comorbidity.

We also found no age-gradient in IADL limitations. There is considerable variability in the way that arthritis-related disability is captured in population based studies [12, 35]. The questions in our study related specifically to difficulties in various IADLs (e.g., bathing or dressing) due to their arthritis, and our estimates were adjusted for a range of other variables including comorbidity. This may partially explain the lack of an age-gradient in our study compared to other studies [12, 35].

Our finding of more arthritis-related sleep difficulties among younger (20–44 years) and middle aged (45–64 years) adults compared to those 75 years and older is very similar to findings from a US population-based survey [36]. While there are very few population based reports of age-related risks of pain or fatigue in individuals with arthritis, we hypothesize that our finding of more frequent and severe pain and fatigue in younger individuals could be linked to their sleep difficulties since pain is a known mediator of sleep problems in individuals with arthritis [36, 37]. Factors such as pain, fatigue and sleep difficulties may also contribute to other aspects of mental health such as anxiety and depression; conversely, an individuals’ perception of pain and fatigue are closely tied to their mental health [38–40]. Further research is needed to better understand how age contributed to the arthritis–related sleep difficulties, and the relationship to other aspects of health and arthritis symptoms.

Our findings related to high levels of stress and worse self-rated mental health in younger (20–44 years) and middle aged (45–64 years) adults with arthritis compared to those 75 years and older is supported by previous population based studies [41, 42]. For instance, data from a US survey shows a decreasing age gradient in the reporting of mental distress among adults with arthritis aged 45 years or older, with the youngest age group (age 45–54) reporting the most distress [41]. Also, data from the US showed individuals with arthritis younger than 65 years, have more anxiety and depression versus those who are older controlling for a range of disease-related factors [42].

Research on the experience of arthritis and its impact on life roles may provide some insight into these findings. A qualitative study found that middle-aged adults reported more distress and frustration compared to those older because their experience of arthritis was not what one would expect at their age or stage in life, was more disruptive of activities/roles related to work and family as well, was threatening to their future plans [43]. Adults in the middle years are often expected to take on a number of diverse roles and if they perceive they cannot participate fully in valued areas of life it may have consequences on psychological health [44]. For instance, researchers have shown that middle-aged adults with OA reported more difficulties with roles than their older counterparts, and found that role limitations and dissatisfaction may contribute to poorer psychological well-being [44]. Studies specifically on employment have shown that many individuals with arthritis report difficulties with work or even have to give up their jobs [45–48]. Moreover, literature of primarily middle age adults has shown that balancing work, health and one’s personal life is stressful for people with arthritis [44, 49, 50].

Our study has a number of strengths including the large population-based sample; the administration of the survey by trained personnel, using a structured format; and data on sleep and IADLs limitations directly attributed to arthritis (versus other co-morbidities). However, the results should be considered in light of several limitations. Firstly, the cross-sectional study design precluded an examination of a potential cohort effect. Older adults may underreport pain because they believe it is a natural part of aging for which they are less likely to seek help [51, 52]. In a study of adults with OA, older primary care patients were more likely than younger patients to believe people should expect to live with pain as they got older [53]. Reporting pain also reflects societal norms regarding stoicism among older adults and the possibility of secondary gain in working age adults. Studies indicate that older adults have an attitude of stoicism leading to a greater reluctance to reporting pain or expressing need [54]. Therefore the higher frequency of reporting recurrent and severe pain among younger versus older individuals could in part be explained by a cohort effect.

Secondly, respondents’ arthritis diagnoses, impact-of-arthritis outcomes and explanatory variables relied on self-report data; therefore, recall and social desirability biases may have resulted in misclassification of results. In addition, the chosen cut-offs for the various impact-of-arthritis outcomes may have resulted in further misclassification of results. However, it is worth noting that self-reported diagnosis of arthritis has been shown to have high reliability, fairly high sensitivity (approaching 84 %) and moderate specificity (71 %) [55, 56]. Also, respondents were asked the case finding question twice, first in the 2008 CCHS interview and then in the 2009 SLCDC interview, providing further confirmation of their arthritis diagnosis [14]. Nevertheless, “arthritis” is a vague term and encompasses more than 100 different types of rheumatic diseases with a wide range of clinical presentations and degrees of impairment [1]. While we controlled for respondents’ type of arthritis, residual confounding may remain due to the fact that self-reported information on type of arthritis (e.g., OA, rheumatoid arthritis etc.) is of questionable validity [57–59] as well, a large proportion of the respondents did not know their type of arthritis (43.2 %).

Lastly, the three to 14 month lag time between the 2008 CCHS and 2009 SLCDC interviews may have affected how current an individual’s body weight was [14], Misclassification of a respondents’ weight is possible; however, this is unlikely to be a significant source of error since the vast majority of individuals do not lose a considerable amount of weight over such a short time frame [60].

## Conclusions

Contrary to the belief that older adults with arthritis experience more severe physical and mental health outcomes, our study demonstrates wide-ranging impacts of arthritis on individuals of all ages. In fact, we found that older adults were less likely to report worse outcomes such as joint pain, sleep limitations, high levels of stress and suboptimal mental health than younger adults. In light of these findings, public health messaging should stress that arthritis does not just affect the elderly [1, 43] and should emphasize the importance of timely diagnosis and treatment at all ages in order to prevent or, minimize arthritis related impairment [1, 61].

## Abbreviations

ACHES: Arthritis Conditions Health Effects Survey
BMI: Body mass index
CCHS: Canadian Community Health Survey
CIs: Confidence intervals
IADLs: Instrumental activities of daily living
NHANES: National Health and Nutrition Examination Survey
NPHS: National Population Health Survey
OA: Osteoarthritis
SAS: Statistical analysis software
US: United States
SLCDC: Survey on Living with Chronic Diseases in Canada
WHO: World Health Organization

## Footnotes

1 The 2008 CCHS used three sampling frames to select the sample of households: 49 % of the sample of households came from an area frame, 50 % came from a list frame of telephone numbers and the remaining 1 % came from a Random Digit Dialling sampling frame. More details regarding the CCHS can be found here: http://www23.statcan.gc.ca/imdb/p2SV.pl?Function=getSurvey&SDDS=3226

2 An Act respecting statistics of Canada: http://laws-lois.justice.gc.ca/eng/acts/S-19/

3 Statistics Canada performed adjustments to the weights of responding persons to account for the loss of excluded or non-responding persons that are part of the SLCDC target population: http://www23.statcan.gc.ca/imdb-bmdi/pub/document/5160_D5_T1_V1-eng.htm
